# Supplementary material for: From sequence to enzyme mechanism using multi-label machine learning
Source: BMC Bioinformatics. 2014 May 19;15:150. doi: 10.1186/1471-2105-15-150 (PMC4229970; doi:10.1186/1471-2105-15-150)
Supplement: Additional file 2 — Java code of ml2db. Additional file ml2db_code.tar.gz contains the Java source code to run the multi-label machine learning experiments and save the results to database. The code’s Javadoc is included. [file 1471-2105-15-150-S2.zip › additional file 2/ml2db/ml2db/doc/main/ml2db/weka/ArffProps.html]

ArffProps


JavaScript is disabled on your browser.


- Overview
- Package
- Class
- Use
- Tree
- Deprecated
- Index
- Help

- Prev Class
- Next Class

- Frames
- No Frames

- All Classes

- Summary:
- Nested |
- Field |
- Constr |
- Method

- Detail:
- Field |
- Constr |
- Method


main.ml2db.weka

## Class ArffProps

- java.lang.Object
- - uk.ac.standrews.utils.main.Initialised
  - - main.ml2db.weka.ArffProps

- All Implemented Interfaces:
  :   uk.ac.standrews.utils.main.Initialisable

  Direct Known Subclasses:
  :   ArffPropsFromFile

  ---

    

  ```
  public class ArffProps
  extends uk.ac.standrews.utils.main.Initialised
  ```

  A class containing the meta-data for a Weka ARFF file (or arff + xml files
  for Mulan ARFF) The data includes the path to an existing ARFF file, an
  optional data set name and description, and various statistics for the data
  (number of instances, class values, attributes)

  Version:
  :   5 Mar 2014

  Author:
  :   Luna De Ferrari ldf@st-andrews.ac.uk

- - ### Field Summary

    Fields

    | Modifier and Type | Field and Description |
    | --- | --- |
    | `static java.lang.String` | `ARFF_DATA_PROP` The full arff data file text taken the ARFF file. |
    | `static java.lang.String` | `ARFF_FILE_PATH_PROP` the file path where the ARFF file will be written, inclusive of file name. |
    | `static java.lang.String` | `CLASS_NAME_PROP` The class name. |
    | `static java.lang.String` | `DATASET_NAME_PROP` the experiment short name |
    | `static java.lang.String` | `DESCRIPTION_PROP` |
    | `static java.lang.String` | `NUMBER_OF_ATTRIBUTES` the total number of possible attributes for the dataset |
    | `static java.lang.String` | `NUMBER_OF_CLASS_VALUES` the total number of possible class values for the dataset |
    | `static int` | `NUMBER_OF_CORE_PROPERTIES` the number of properties that should be available on the properties file (check) |
    | `static java.lang.String` | `NUMBER_OF_INSTANCES` the number of instances (rows) in the arff file |
    | `static int` | `NUMBER_OF_STATISTICS_PROPERTIES` the number of properties + number of data statistics (check: should be 9 + 3) |
    | `static java.lang.String` | `XML_DATA_PROP` |
    | `static java.lang.String` | `XML_FILE_PATH_PROP` needed for compatibility with Mulan arff files (which have 1 arff file + 1 xml labels file) |

    - ### Fields inherited from class uk.ac.standrews.utils.main.Initialised

      `m_initialisationIsCorrect`
  - ### Constructor Summary

    Constructors

    Constructor and Description

    `ArffProps(java.lang.String arffFilePath,
    java.lang.String xmlFilePath)`

    Xml path is there for compatibility with Mulan, can be left null or
    empty.

    `ArffProps(java.lang.String arffFilePath,
    java.lang.String xmlFilePath,
    java.lang.String datasetDescription)`

    A textual, human readable description for the data
  - ### Method Summary

    Methods

    | Modifier and Type | Method and Description |
    | --- | --- |
    | `boolean` | `checkPropertiesKeys(java.util.Properties props)` |
    | `java.util.TreeSet<java.lang.String>` | `coreKeys()` The core ARFF properties, essential to load the data from a Weka ARFF file |
    | `boolean` | `corePropertiesAreCorrect(java.util.Properties props)` |
    | `java.lang.String` | `deriveXmlPath()` |
    | `java.lang.String` | `getArffFilePath()` Get the path to the ARFF file |
    | `uk.ac.standrews.utils.main.database.DbConn` | `getDbConn()` |
    | `int` | `getNumberOfCoreProperties()` |
    | `int` | `getNumberOfStatsProperties()` |
    | `java.util.Properties` | `getProperties()` Get the properties |
    | `java.lang.String` | `getProperty(java.lang.String propertyName)` Get a property value |
    | `java.lang.String` | `getXmlFilePath()` Get the path to the Mulan XML file |
    | `boolean` | `propertiesAreCorrect(java.util.Properties props)` |
    | `java.util.TreeSet<java.lang.String>` | `propertiesKeys()` All properties: core + statistics |
    | `void` | `saveStatisticsAsProperties(java.lang.String numberOfAttributes, java.lang.String numberOfClassValues, java.lang.String numberOfInstances)` |
    | `java.util.TreeSet<java.lang.String>` | `statisticsKeys()` The data statistics properties (number of attributes, classes, instances) added after the arff file generation |
    | `boolean` | `statsPropertiesAreCorrect(java.util.Properties props)` |

    - ### Methods inherited from class uk.ac.standrews.utils.main.Initialised

      `initialisationIsCorrect, setInitialisation, setInitialisationIsCorrect, setInitialisationNotCorrect`
    - ### Methods inherited from class java.lang.Object

      `equals, getClass, hashCode, notify, notifyAll, toString, wait, wait, wait`

- - ### Field Detail


    - #### ARFF\_FILE\_PATH\_PROP

      ```
      public static final java.lang.String ARFF_FILE_PATH_PROP
      ```

      the file path where the ARFF file will be written, inclusive of file
      name. A timestamp and the .arff ending will be added automatically.

      See Also:
      :   Constant Field Values


    - #### XML\_FILE\_PATH\_PROP

      ```
      public static final java.lang.String XML_FILE_PATH_PROP
      ```

      needed for compatibility with Mulan arff files (which have 1 arff file +
      1 xml labels file)

      See Also:
      :   Constant Field Values


    - #### ARFF\_DATA\_PROP

      ```
      public static final java.lang.String ARFF_DATA_PROP
      ```

      The full arff data file text taken the ARFF file.

      See Also:
      :   Constant Field Values


    - #### XML\_DATA\_PROP

      ```
      public static final java.lang.String XML_DATA_PROP
      ```

      See Also:
      :   Constant Field Values


    - #### CLASS\_NAME\_PROP

      ```
      public static final java.lang.String CLASS_NAME_PROP
      ```

      The class name. Examples, for a medical prediction: has\_disease.

      See Also:
      :   Constant Field Values


    - #### DATASET\_NAME\_PROP

      ```
      public static final java.lang.String DATASET_NAME_PROP
      ```

      the experiment short name

      See Also:
      :   Constant Field Values


    - #### DESCRIPTION\_PROP

      ```
      public static final java.lang.String DESCRIPTION_PROP
      ```

      See Also:
      :   Constant Field Values


    - #### NUMBER\_OF\_ATTRIBUTES

      ```
      public static final java.lang.String NUMBER_OF_ATTRIBUTES
      ```

      the total number of possible attributes for the dataset

      See Also:
      :   Constant Field Values


    - #### NUMBER\_OF\_CLASS\_VALUES

      ```
      public static final java.lang.String NUMBER_OF_CLASS_VALUES
      ```

      the total number of possible class values for the dataset

      See Also:
      :   Constant Field Values


    - #### NUMBER\_OF\_INSTANCES

      ```
      public static final java.lang.String NUMBER_OF_INSTANCES
      ```

      the number of instances (rows) in the arff file

      See Also:
      :   Constant Field Values


    - #### NUMBER\_OF\_CORE\_PROPERTIES

      ```
      public static final int NUMBER_OF_CORE_PROPERTIES
      ```

      the number of properties that should be available on the properties file
      (check)

      See Also:
      :   Constant Field Values


    - #### NUMBER\_OF\_STATISTICS\_PROPERTIES

      ```
      public static final int NUMBER_OF_STATISTICS_PROPERTIES
      ```

      the number of properties + number of data statistics (check: should be 9
      + 3)

      See Also:
      :   Constant Field Values
  - ### Constructor Detail


    - #### ArffProps

      ```
      public ArffProps(java.lang.String arffFilePath,
               java.lang.String xmlFilePath)
      ```

      Xml path is there for compatibility with Mulan, can be left null or
      empty.

      Parameters:
      :   `arffFilePath` - the path to the weka arff file
      :   `xmlFilePath` - Xml file path is for compatibility with Mulan, can be left
          null or empty.


    - #### ArffProps

      ```
      public ArffProps(java.lang.String arffFilePath,
               java.lang.String xmlFilePath,
               java.lang.String datasetDescription)
      ```

      A textual, human readable description for the data
  - ### Method Detail


    - #### checkPropertiesKeys

      ```
      public boolean checkPropertiesKeys(java.util.Properties props)
      ```


    - #### coreKeys

      ```
      public java.util.TreeSet<java.lang.String> coreKeys()
      ```

      The core ARFF properties, essential to load the data from a Weka ARFF
      file

      Returns:
      :   the essential properties to to load the data from a Weka ARFF
          file


    - #### corePropertiesAreCorrect

      ```
      public boolean corePropertiesAreCorrect(java.util.Properties props)
      ```


    - #### getArffFilePath

      ```
      public java.lang.String getArffFilePath()
      ```

      Get the path to the ARFF file

      Returns:
      :   the path to the ARFF file


    - #### getXmlFilePath

      ```
      public java.lang.String getXmlFilePath()
      ```

      Get the path to the Mulan XML file

      Returns:
      :   the path to the Mulan XML file


    - #### getDbConn

      ```
      public uk.ac.standrews.utils.main.database.DbConn getDbConn()
      ```


    - #### getNumberOfCoreProperties

      ```
      public int getNumberOfCoreProperties()
      ```


    - #### getNumberOfStatsProperties

      ```
      public int getNumberOfStatsProperties()
      ```


    - #### getProperties

      ```
      public java.util.Properties getProperties()
      ```

      Get the properties


    - #### getProperty

      ```
      public java.lang.String getProperty(java.lang.String propertyName)
      ```

      Get a property value

      Parameters:
      :   `propertyName` - the name of the property (key)

      Returns:
      :   the associated property value


    - #### deriveXmlPath

      ```
      public java.lang.String deriveXmlPath()
      ```


    - #### propertiesAreCorrect

      ```
      public boolean propertiesAreCorrect(java.util.Properties props)
      ```


    - #### propertiesKeys

      ```
      public java.util.TreeSet<java.lang.String> propertiesKeys()
      ```

      All properties: core + statistics

      Returns:


    - #### saveStatisticsAsProperties

      ```
      public void saveStatisticsAsProperties(java.lang.String numberOfAttributes,
                                    java.lang.String numberOfClassValues,
                                    java.lang.String numberOfInstances)
      ```


    - #### statisticsKeys

      ```
      public java.util.TreeSet<java.lang.String> statisticsKeys()
      ```

      The data statistics properties (number of attributes, classes, instances)
      added after the arff file generation

      Returns:


    - #### statsPropertiesAreCorrect

      ```
      public boolean statsPropertiesAreCorrect(java.util.Properties props)
      ```


- Overview
- Package
- Class
- Use
- Tree
- Deprecated
- Index
- Help

- Prev Class
- Next Class

- Frames
- No Frames

- All Classes

- Summary:
- Nested |
- Field |
- Constr |
- Method

- Detail:
- Field |
- Constr |
- Method
